# Al-Coated Conductive Fiber Filters for High-Efficiency Electrostatic Filtration: Effects of Electrical and Fiber Structural Properties

**DOI:** 10.1038/s41598-018-23960-9

**Published:** 2018-04-10

**Authors:** Dong Yun Choi, Eun Jeong An, Soo-Ho Jung, Dong Keun Song, Yong Suk Oh, Hyung Woo Lee, Hye Moon Lee

**Affiliations:** 1 Powder Technology Department, Powder & Ceramics Division, Korea Institute of Materials and Science, Changwon, 51508 Republic of Korea; 2 Department of Nano Fusion Technology, Pusan National University, Busan, 46241 Republic of Korea; 3 Department of Environmental Machinery, Environment System Research Division, Korea Institute of Machinery and Materials, Daejeon, 34103 Republic of Korea; 4 Department of Mechanical Engineering, Korea Advanced Institute of Science and Technology, Daejeon, 34141 Republic of Korea; 5 Alink Co. Ltd., Changwon, 51508 Republic of Korea

## Abstract

Through the direct decomposition of an Al precursor ink AlH_3_{O(C_4_H_9_)_2_}, we fabricated an Al-coated conductive fiber filter for the efficient electrostatic removal of airborne particles (>99%) with a low pressure drop (~several Pascals). The effects of the electrical and structural properties of the filters were investigated in terms of collection efficiency, pressure drop, and particle deposition behavior. The collection efficiency did not show a significant correlation with the extent of electrical conductivity, as the filter is electrostatically charged by the metallic Al layers forming electrical networks throughout the fibers. Most of the charged particles were collected via surface filtration by Coulombic interactions; consequently, the filter thickness had little effect on the collection efficiency. Based on simulations of various fiber structures, we found that surface filtration can transition to depth filtration depending on the extent of interfiber distance. Therefore, the effects of structural characteristics on collection efficiency varied depending on the degree of the fiber packing density. This study will offer valuable information pertaining to the development of a conductive metal/polymer composite air filter for an energy-efficient and high-performance electrostatic filtration system.

## Introduction

Currently, great concern over the removal of airborne particulate matter (PM) has fostered the development of air filtration technologies to improve air quality levels. Specifically, nanosized PM, which can penetrate the human bronchi and lungs, has been proven to pose negative health effects such as cardiovascular and respiratory diseases^1–4^. Moreover, PM suspended in air has adverse effects on our living environment in terms of visibility, radiative force, and climate change^5–8^.

Fibrous air filters constructed with random networks of microscale and/or nanoscale fibers are applied to various air filtration systems because they are inexpensive, light, and simple to install. However, in normal fibrous filters, it is difficult to improve the PM collection efficiency while maintaining a pressure drop across the filter due to an existing trade-off relationship between these factors. Because the pressure drop is directly proportional to the energy consumption of a fan^9,10^, it is crucial to enhance the PM collection efficiency without increasing the pressure drop.

Numerous efforts have been made to address the above issue. Examples include electret filters with electrostatic effects arising from quasi-permanent electrical charges on dielectric polymer fibers^11–14^, nanofiber filters made of highly polar polymers showing high binding affinity to PM^15–17^, and fibrous filters hybridized with carbon nanotubes (CNTs) exhibiting the slip flow effect at the CNT surfaces^18,19^. These novel approaches suggest physicochemical structures of filter fibers capable of improving collection efficiency without a severe degradation in air permeability, but they still require a relatively high packing density of the fibers to achieve a collection efficiency exceeding 99%. Consequently, they also involve pressure drops in the range of several tens to several hundreds of Pascals.

Electrostatic precipitators (ESPs) based on Coulombic attractions between charged particles and a charged collection plate have been widely adopted industrially to remove the PM suspended in exhaust gas owing to their extremely low pressure drop and high collection efficiency^20,21^. If a fibrous filter has high electrical conductivity, the ESP technology can be applied directly to the filter. Recently, our group introduced an Al-coated conductive fiber (ALCF) filter fabricated by the reaction of the direct decomposition of Al onto the fibers of a polyester (PET) nonwoven filter using an Al precursor ink AlH_3_{O(C_4_H_9_)_2_}^22^. The ALCF filter yielded high electrostatic PM removal rates of >99% for particles sizes ranging from 30 to 410 nm while showing low pressure drops in the region of ~4.9 Pa at an airflow velocity of 10 cm/s. Compared to electret filters, the extent of charges and the electric field strength of the fibers could be easily controlled using an external high-voltage device owing to the excellent conductivity of the ALCF filter.

Numerous electrostatic filtration characteristics regarding particle penetration, particle loading behavior, and the effects of the physical and electrical properties of the particles, for instance, have been analyzed^11–13^. However, these studies focused mainly on nonconductive filters electrostatically reinforced by charge injection^23,24^ and/or by the polarization of filter fibers under external electric fields^25–27^. Although there have been several conductive filters consisting of a stainless steel (SUS) mesh^28^ and silver nanowire percolation network^29^, the effects of the electrical and structural properties of conductive filter media have yet to be examined with regard to the electrostatic filtration performance capabilities. Deep insight into the above matters would be very helpful in realizing high-performance conductive fiber filters with a highly cost-effective approach.

In the present work, we prepared various ALCF filters with different fiber structural properties, specifically the thickness, porosity, and fiber size, via a simple solution-dipping (SD) process based on an Al precursor ink AlH_3_{O(C_4_H_9_)_2_}. We investigated the electrostatic filtration performance, including particle collection efficiency, pressure drop, and quality factor, of the ALCF filters in terms of their electrical and structural properties. We experimentally confirmed that particle collection by electrostatic interactions in the ALCF filter mainly occurs on the exterior filter surfaces, where the electric potential gradient is high. Through numerical simulations of particle motion, we verified the surface filtration of the charged particles under a Coulombic force. In addition, we numerically analyzed the effects of fiber size and packing density on collection efficiency, quality factor, and particle deposition behavior.

## Results

### Fabrication of the ALCF filter

**Figure 1:**
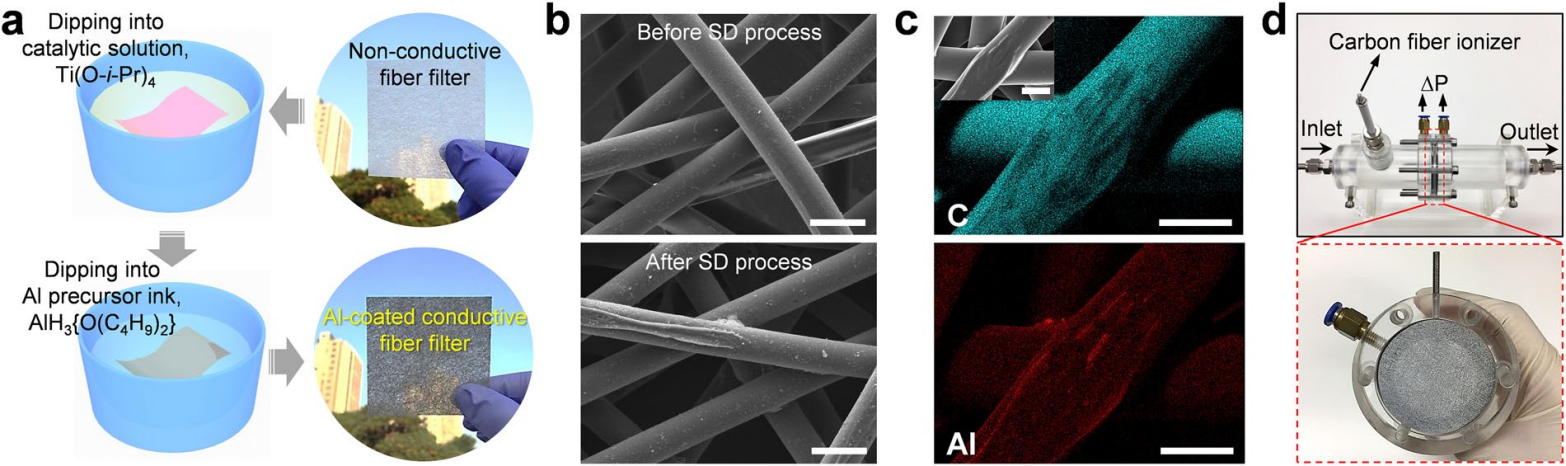
(**a**) Sketch of the solution-dipping (SD) process used to fabricate the Al-coated conductive fiber (ALCF) filters. (**b**) SEM images of a raw PET filter (top) and an ALCF filter (bottom). The scale bars are 50 μm. (**c**) EDS mapping results of ALCF filter for carbon (C; cyan dots) and aluminum (Al; red dots). The inset presents a magnified image of the microstructure of an ALCF filter. The scale bars denote 30 μm. (**d**) Photographs of the assembled device (top) and the ALCF filter loaded onto a plastic holder (bottom).

The ALCF filters were fabricated using commercially available PET nonwoven fabrics via a SD process. Figure 1a presents a schematic diagram of the SD process that involves two steps: (i) a catalytic treatment by titanium isopropoxide (Ti(O-*i*-Pr)_4_) and (ii) dip-coating into the Al precursor ink AlH_3_{O(C_4_H_9_)_2_} chemically synthesized by an ethereal reaction of aluminum chloride (AlCl_3_) with lithium aluminum hydride (LiAlH_4_) in dibutyl ether (O(C_4_H_9_)_2_). More detailed fabrication procedures for the Al precursor ink and ALCF filter are explained in our previous work^22,30^. The top-right and bottom-right images shown in Fig. 1a correspond to the filter before and after the SD process, respectively. The formation of Al features on fibers could be identified with the naked eye by the color change of the filter from white to metallic gray. The scanning electron microscopy (SEM) images shown in Fig. 1b reveal that the microstructure of a pristine filter retained its integrity after the SD process. Figure 1c shows an energy-dispersive spectroscopy (EDS) mapping of the ALCF filter, indicating that both carbon (cyan dots) and aluminum (red dots) are evenly distributed over the fiber surface. Hence, these results demonstrate the densely structured Al features formed on the fibers by the SD process.

Figure S1 displays a schematic of the electrostatic filtration device used in this study. The device is composed of a carbon fiber ionizer generating unipolar ionized air and a single ALCF filter collecting incoming charged particles. The ionizer was positioned at a location approximately 50 mm ahead of the ALCF filter, and its electric potential (*V*_i_) was controlled using a negative high-voltage generator. To control the electric potential of the ALCF filter (*V*_f_), the filter was tightly attached to a SUS electrode connected to a positive high-voltage generator. The inlet and outlet SUS tubes of the device were grounded for all filtration tests, and the outlet was positioned 80 mm downstream of the filter. Figure 1d shows images of the filtration device (top) and the ALCF filter positioned on the SUS electrode (bottom).

### Effect of the electrical resistance

**Figure 2:**
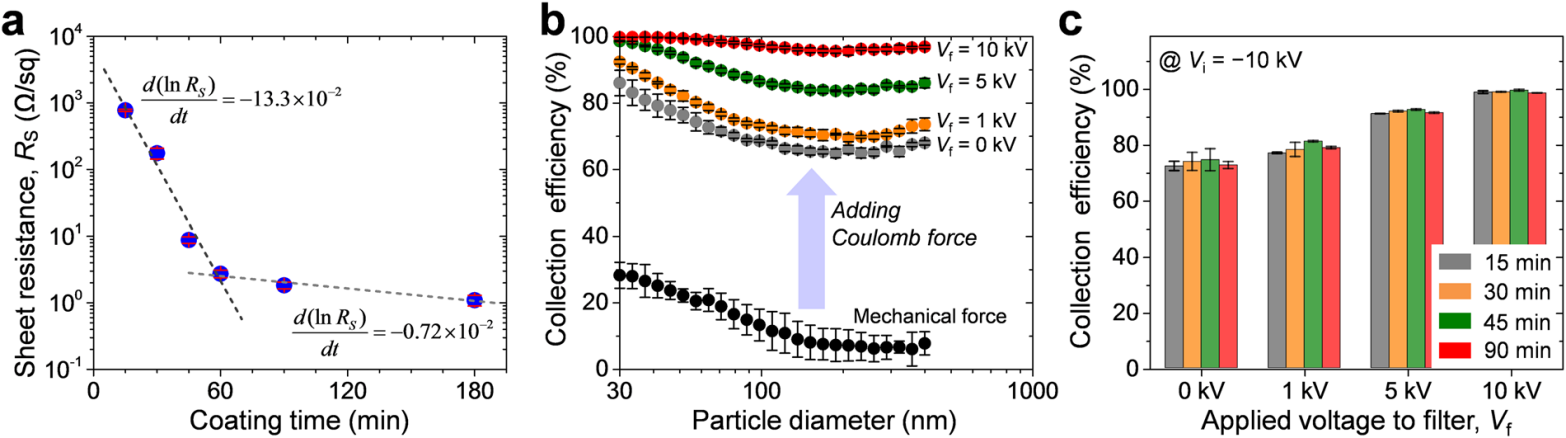
(**a**) Variation of the sheet resistances of ALCF filters as a function of immersion time in the Al precursor ink. (**b**) The collection efficiency profiles of ALCF filters as a function of particle diameter. (**c**) Bar graph comparing the collection efficiencies of ALCF filters prepared for different coating times (15, 30, 45, and 90 min). The height of a bar corresponds to the average collection efficiency for particles in the size range 30–410 nm.

The conductivity of the ALCF filter was controlled simply by varying the immersion time in the Al precursor ink, and the resistance values were plotted as a function of the coating time, as shown in Fig. 2a. The sheet resistance (*R*_S_) exponentially decreased with increasing coating time, and the rate of resistance decrease differed near 60 min. The ALCF filter showed a high *R*_S_ of ~770 Ω sq^−1^ at 15 min, though this value dropped to ~2.7 Ω sq^−1^ after 60 min. As time elapsed, the *R*_S_ decreased more slowly, eventually reaching ~1.1 Ω sq^−1^ at 180 min. Note that we were not able to obtain ALCF filters with a stable and reliable electrical property at a coating time of less than 15 min.

The particle collection efficiency was examined using potassium chloride (KCl) nanoparticles in the size range 30–410 nm. The size distribution of the generated KCl particles is depicted in Fig. S2, and their geometric mean diameter (GMD) and geometric standard deviation (GSD) were 70.1 nm and 1.86, respectively. The collection efficiency was calculated as,

1 $$\eta =1-\frac{{C}_{down}}{{C}_{up}}.$$

Here, *C*_up_ and *C*_down_ represent the particle concentrations (particles/cm^3^) upstream and downstream of the filter, respectively. Figure S3 presents a schematic of the experimental setup, and the detailed measurement procedures are described in the Method section. Figure 2b shows the fractional collection efficiency of an ALCF filter prepared with a coating time of 30 min. When the electrostatic effect was absent (*V*_i_ & *V*_f_ = 0 kV), the collection efficiency by a mechanical filtration mechanism, such as inertial impaction, interception, and Brownian diffusion, increased from ~5% to ~28% as the particle size decreased (black circles). When electric fields were established between the ALCF filter (*V*_f_ = 0 kV) and the ionizer (*V*_i_ = −10 kV), the charged particles were collected with considerably improved efficiency (more than three-fold) under the action of Coulomb force. Moreover, further enhancement of the electric field strength by the increase in *V*_f_ progressively improved the collection efficiency to ~ 99%.

To investigate the effect of the electrical properties of the ALCF filters on the removal performance of PM, we compared the collection efficiencies of filters prepared at different coating times 15, 30, 45, and 90 min. Figure 2c presents the average collection efficiency for particles in the size range 30–410 nm as a function of *V*_f_ when *V*_i_ was held at −10 kV. The collection efficiency of each filter improved as *V*_f_ was increased. However, the efficiency at a certain *V*_f_ was similar regardless of the level of resistance, because the surface potential of the fiber surface is not dependent on the metal film thickness. That is, the ALCF filter can be sufficiently electrostatically charged to produce a strong electric field by an external high-voltage source once conductive networks are constructed by the metallic Al features formed on every unit fiber. Thus, these results suggest that the performance of the ALCF filter is hardly affected by the thickness of the Al layer coated over the fibers, suggesting that a high-performance ALCF filter can be realized more economically by using less Al.

### Effect of filter thickness

**Figure 3:**
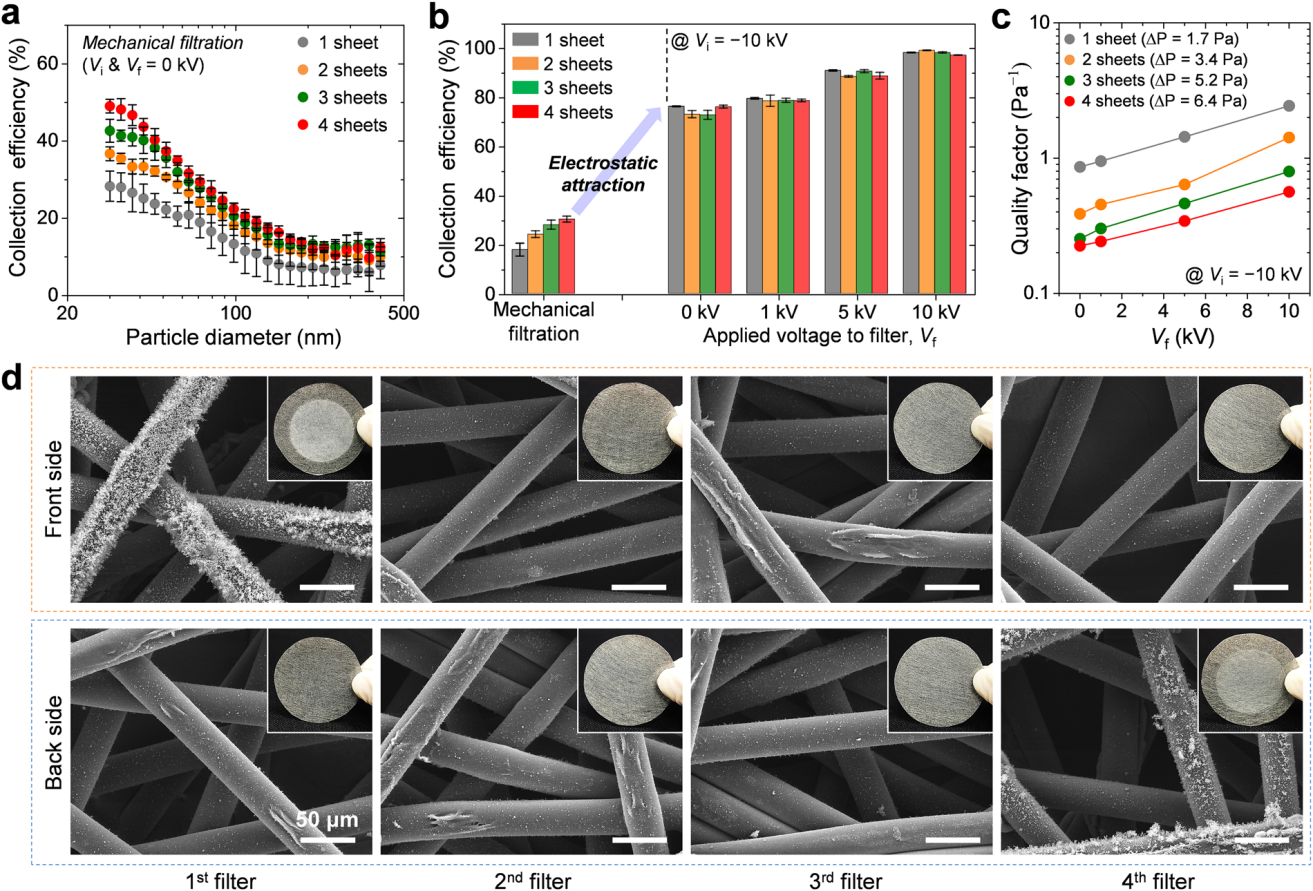
(**a**) Profiles of the mechanical collection efficiency according to the number of filter sheets overlapped with each other (*V*_i_ & *V*_f_ = 0). (**b**) Bar graph comparing the average collection efficiencies according to the number of sheets at various values of *V*_f_. (**c**) Plot of the quality factor as a function of *V*_f_ for different number of sheets. (**d**) SEM images presenting the front and back surfaces of each ALCF filter after a filtration experiment involving four stacked filters. Each inset photograph shows the corresponding surface of the ALCF filter. The scale bars are 50 μm.

In general, particle penetration, which is expressed as *ϕ* = exp(−4*αη*_*s*_*t*_f_/(π*d*_f_(1 − *α*))), decreases exponentially as the filter is thickened^31^. Here, *α* is the fiber volume fraction, *η*_*s*_ is the single-fiber efficiency, *t*_f_ is the filter thickness, and *d*_f_ is the fiber diameter. To determine the influence of filter thickness on the electrostatic collection performance of the ALCF filter, we increased the overall thickness by overlapping several filters with each other. Figure 3a shows the changes in the collection efficiency profiles according to the number of filter sheets when the mechanical filtration mechanism was the main function (*V*_i_ and *V*_f_ = 0 kV). As expected, the efficiency increased with the number of sheets. The degree of improvement was more noticeable below 100 nm size owing to the diffusion force.

The bar graph in Fig. 3b shows the results of comparison between the average electrostatic collection efficiency and mechanical collection efficiency for particles in the size range 30–410 nm. Whereas the performance by mechanical means was gradually improved with an increase in the filter thickness, the charged particles were captured with very similar efficiencies, irrespective of the number of overlapped filters, for each *V*_f_ condition. Considering the Al layers wrapping the PET fibers have identical electric potential under electrostatic conditions, the electric potential gradient inside the ALCF filter is negligible, but the gradient near the outer surface of the filter can be large enough to form a strong electric field. Thus, most of the charged particles are captured by the surface filtration based on Coulombic interactions, which results in a negligible influence of filter thickness on collection efficiency.

Therefore, taking into account the pressure drop (Δ*P*) across all filters, the use of a single filter is more desirable for improving the quality factor, which is defined as QF = −ln(*ϕ*)/Δ*P*, as shown in Fig. 3c. The QF value increased linearly on a logarithmic scale as *V*_f_ was increased, and the QF curve moved parallel to the *y*-direction as the number of filter sheets was decreased. Because the increase in the filter thickness increased not the collection efficiency but the pressure drop, an increase in the number of filters simply led to a degradation of the quality factor.

Figure 3d shows the surface analysis results of each sheet after a filtration test of four filter sheets overlapped with each other. The rows correspond to the front and back side of the sheet, and the columns represent the order of the filters installed in the device; the first and fourth filters were close to the upstream and downstream sides, respectively. At the front side of the first filter and the back side of the fourth filter, the white dust of the captured KCl particles is clearly distinguishable from the metal gray surface of the ALCF filter, as shown in the inset photographs in Fig. 3d. However, the deposited KCl particles were difficult to detect by naked eye examination of the surfaces of the other filters. The electric potential gradient inside the layered ALCF filters is relatively negligible, but the potential gradient at the external surfaces of the layered ALCF filters can be strong enough to enable the effective electrostatic capture of the charged particles. Hence, the incoming particles are preferentially deposited on the front side of the first filter facing the carbon fiber ionizer, and some particles penetrating the ALCF filters are collected on the back side of the fourth filter facing the electrically grounded outlet SUS tube. From SEM analysis, it was found that the electrostatic capturing process was effective until the third fiber layer for the front side of the first filter and the back side of the fourth filter, indicating that the charged particles were mainly captured on the outer surface of the overlapped filters where high electric fields had formed.

### Effect of structural properties of the filter

To investigate the influence of structural properties of the filter, such as the fiber diameter, porosity, and thickness, on PM removal performance, we selected four different types of PET nonwoven filters. The microstructures of these PET filters before and after the SD process are shown in the SEM images in Fig. S4, and the structural properties of these filters are summarized in Table 1. ALCF-1 and ALCF-2 have an effective fiber diameter of approximately 34 μm, and the diameters of ALCF-3 and ALCF-4 are approximately 22 μm. ALCF-1 is constructed with more fibers and its thickness and packing density levels are greater than those of ALCF-2. Similarly, the thickness and packing density of ALCF-3 are higher than those of ALCF-4.

**Table 1:** Summary of the physical properties of the filter samples.

Sample	Areal mass^a^ (g/m^2^)	Effective fiber diameter^b^ (μm)	Thickness (μm)	Porosity (%)	Pressure drop (Pa) @ 5 cm/s
ALCF-1	70	34.2	250 ± 7.1^c^	79.0 ± 3.4	1.7 ± 0.3
ALCF-2	50	34.0	208 ± 8.4	81.9 ± 3.8	1.0 ± 0.2
ALCF-3	60	22.3	124 ± 11.4	74.9 ± 1.7	3.2 ± 0.4
ALCF-4	20	22.2	68 ± 8.4	81.6 ± 0.7	0.8 ± 0.1

^a^Product specification provided by the material supplier (Daekyung Nonwovens Co., Ltd.).

^b^As determined using the empirical relation given by Davies^36^: ∆*P* = *μt*_f_*u*{64*α*^1.5^(1 + 56*α*^3^)}/*d*_f_^2^, where *μ* and *u* are the viscosity and velocity of air, respectively.

^c^Average ± standard deviation.

**Figure 4:**
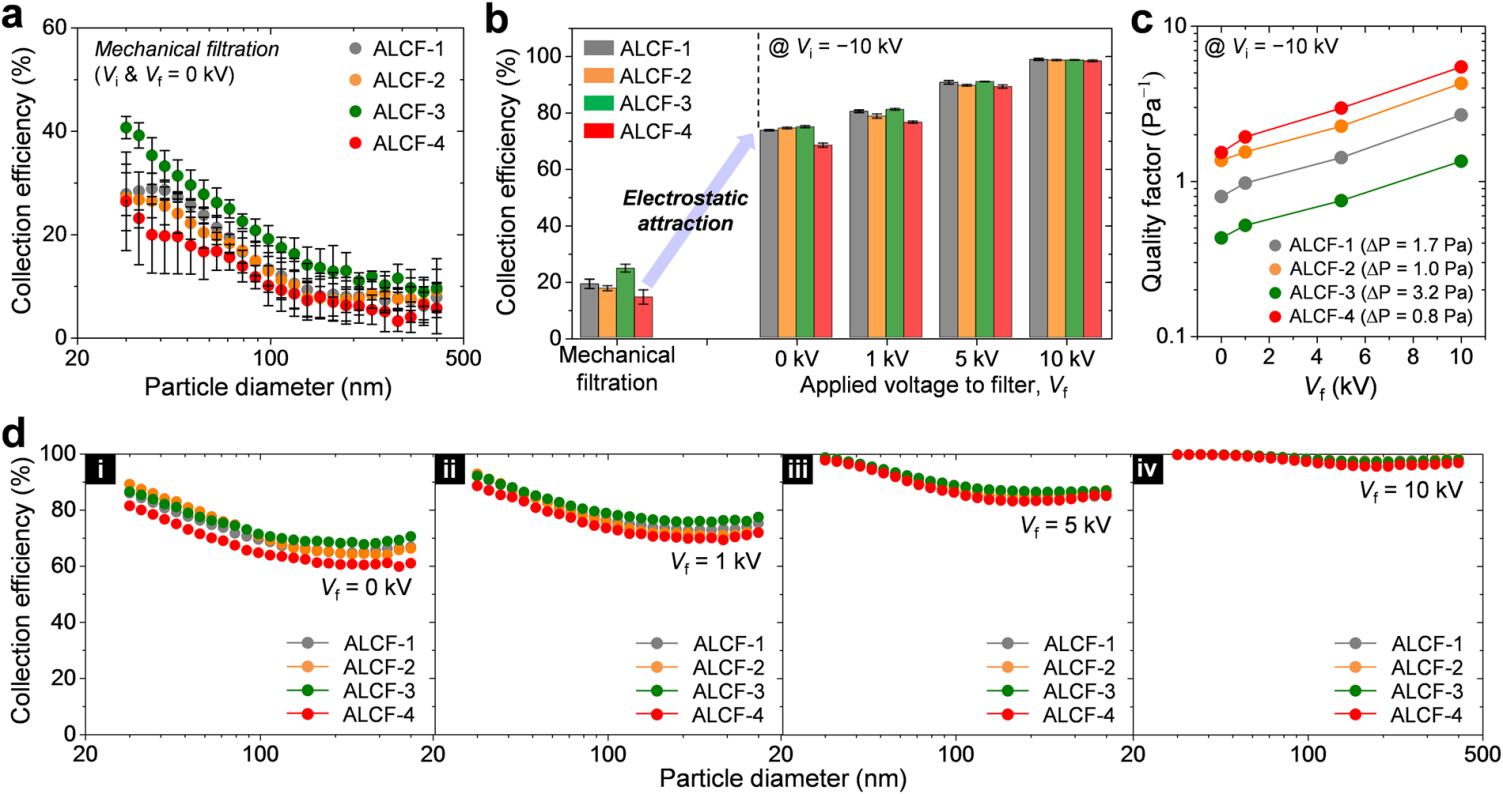
(**a**) Profiles of the mechanical collection efficiencies of ALCF filters with different fiber structures (*V*_i_ & *V*_f_ = 0). (**b**) Bar graph comparing the average collection efficiencies among the four different ALCF filters under different electrostatic field conditions. (**c**) Plot of the quality factor as a function of *V*_f_ for different filter types. (**d**) Comparison of the collection efficiency profiles of each ALCF filter. *V*_i_ was fixed at −10 kV, and *V*_f_ had values of (i) 0 kV, (ii) 1 kV, (iii) 5 kV, and (iv) 10 kV.

Figure 4a presents the collection efficiency of each sample according to particle size when mechanical filtration is the main operating mechanism (*V*_i_ & *V*_f_ = 0 kV). The efficiency profiles exhibited similar shapes for all filters, and the collection efficiencies ranked in decreasing order as follows: ALCF-3, ALCF-1, ALCF-2, and ALCF-4. Although ALCF-3 was thinner than ALCF-1, the higher collection efficiency of ALCF-3 was ascribed to its small fiber size and high packing density. The thinnest ALCF-4 showed the lowest collection efficiency, although its single-fiber efficiency was better than that of ALCF-2.

The bar graph in Fig. 4b compares the average electrostatic collection efficiency and mechanical collection efficiency outcomes. When charged particles passed through the grounded filter (*V*_i_ = −10 kV and *V*_f_ = 0 kV), the collection efficiencies for these filters were improved by more than 3.5 times owing to the action of Coulombic interactions. Similar to the case of mechanical filtration, ALCF-3 showed the highest collection efficiency, while the performance of ALCF-4 was the lowest, which reflects the influence of the mechanical filtration under a relatively weak electric field. However, the difference in their removal performance narrowed as the electric fields became stronger, and the influence of the physical structure on the electrostatic collection efficiency was no longer significant at *V*_f_ ≥ 5 kV.

In terms of pressure drop, the quality factor of ALCF-4 was superior to those of the other filters for all *V*_f_ conditions (Fig. 4c). These results were analogous to the trend shown in Fig. 3c; i.e., the filter thickness does not critically affect the electrostatic PM removal performance of our ALCF filters because the surface filtration process is dominant relative to the depth filtration process. For the ALCF filters used in this study, their structural properties did not have a significant effect on the differences in the collection efficiency compared to their pressure drops, and thus a filter with high air permeability could offer a higher quality factor.

Figure 4d depicts the collection efficiency curves of each filter according to particle size. Each curve shows a similar shape regardless of the structural characteristics. The differences in the fractional efficiencies of these filters were somewhat identified as generally below *V*_f_ = 1 kV, but they became almost indistinguishable above *V*_f_ = 5 kV.

### Numerical simulation of particle collection characteristics

**Figure 5:**
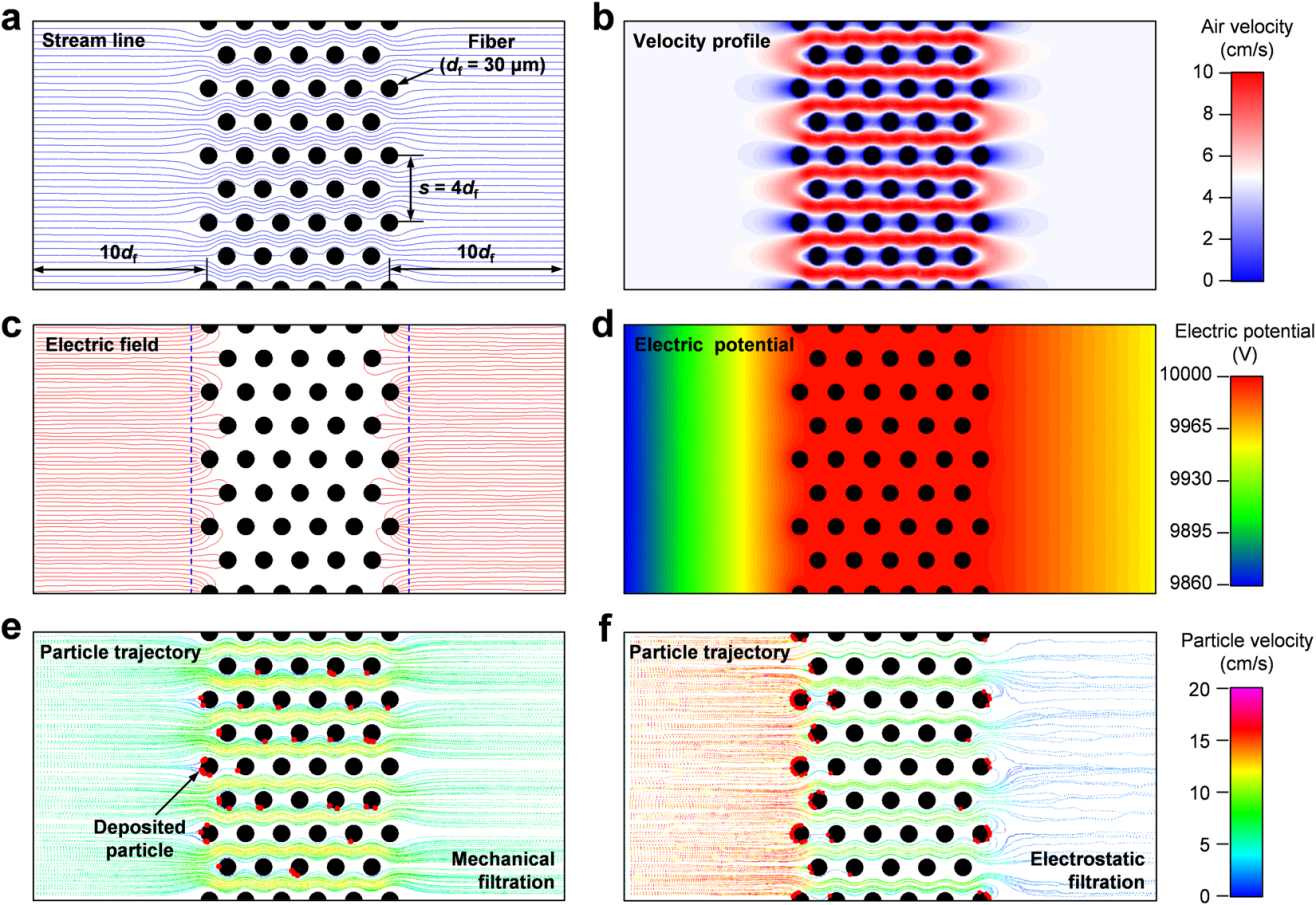
Numerical simulations of the flow field, electric field, and particle motion around staggered fibers with a fiber diameter of 30 μm and interfiber spacing (*s*) of 4*d*_f_. The air flows from left to right and its inlet velocity is 5 cm/s. (**a**) Stream lines and corresponding (**b**) velocity profile. (**c**) Electric field lines produced ahead of the outermost fibers and the corresponding (**d**) electric potential profile. Each fiber has an electric potential of 10 kV. (**e**) Particle trajectories when the Coulomb force does not act on particles with a diameter (*d*_p_) of 0.3 μm. (**f**) Particle trajectories for charged particles (*d*_p_ = 0.3 μm) passing around fibers where electrostatic fields are formed.

Thus far, we have shown that the main filtration mechanism in our ALCF filters is the surface filtration process through Coulomb forces acting on charged particles under electric fields formed in the vicinity of fibers on the exterior of the filter. To simulate these collection behaviors of charged particles by electrostatic forces, the ALCF filter was modeled as a filter consisting of regularly staggered fibers, as shown in Fig. 5a. A 2D model was constructed with eleven fiber layers, and the interfiber spacing in vertical direction (*s*) was set to 4*d*_f_. Thus, the fiber packing density was *α* = *π*${d}_{{\rm{f}}}^{2}$/(4*sd*_f_) ≈ 19.6%. To assign the number of charges on a particle (*n*), we examined the particle charging characteristics depending on the magnitudes of *V*_i_ and *V*_f_. Figure S5a shows the current (*I*) curves as a function of the electric field strength (*E*) between the ionizer and the filter. The ion concentration (*N*_i_) was calculated from the measured *I*, as follows^32^:

2 $${N}_{i}=\frac{I}{A{Z}_{i}Ee},$$

where *A* is the filter area, *Z*_i_ is the ion mobility, and *e* is the elementary charge. The values of *n* on a particle with a diameter (*d*_p_) of 0.3 μm were obtained using the combined charging rate model proposed by Lawless^33^, which simultaneously takes into account diffusion charging and field charging. The calculated results of *N*_i_ and *n* are plotted as a function of *E* in Fig. S5b,c, respectively. In all of the calculations, the particle charge was assigned a value of ~6.0 × 10^−18^ C, corresponding to the conditions of *V*_i_ = −10 kV and *V*_f_ = 10 kV. The electric potential of each fiber surface was set to 10 kV. The mean electric field strengths for the upstream and downstream sides of the filter were assumed to be 4 kV/cm and 1.25 kV/cm, respectively.

Figure 5a shows the symmetric and wavy streamlines formed inside the fiber layers for an inlet air velocity of 5 cm s^−1^; the corresponding velocity profile is displayed in Fig. 5b. The electric field lines formed at the positions depicted by dashed lines ahead of the outermost fibers are presented in Fig. 5c. Note that for clear visualization we only drew the electric field lines passing through the selected points, which are evenly spaced on the dashed line. The electric field lines are concentrated around the first and second fiber layers, whereas they did not develop well inside the fiber layers because the electric potential gradient was relatively negligible, as shown in Fig. 5d. The simulated particle trajectories in Fig. 5e,f compare the deposition behavior of the particles (*d*_p_ = 0.3 μm) depending on the electrostatic effect. When only mechanical forces are present (Fig. 5e), the majority of the particles follows the gas streamlines well, and some particles, marked with red spots, are deposited on the inside fibers as well as the outer fibers, indicating the depth filtration process. As shown in Fig. 5f, once the Coulomb forces are established, however, the particle motions significantly deviate from the gas streamline and turn toward the outermost fiber surfaces. This simulated particle deposition behavior was fairly consistent with the earlier experimental results pertaining to the surface filtration.

In addition, we conducted numerical analyses of the particle deposition behavior, collection efficiency, and pressure drop for various fiber structures with different fiber sizes and packing densities. The fiber diameters were 10, 20, 30, and 40 μm, and the interfiber spacing values for each fiber size were set to 2*d*_f_, 3*d*_f_, 5*d*_f_, 7*d*_f_, and 10*d*_f_. The resulting porosity values were approximately 60.7%, 73.8%, 84.3%, 88.8%, and 92.1%, respectively. Note that eleven fiber layers were considered in all calculations; hence, the filter thickness values increased linearly with fiber size.

**Figure 6:**
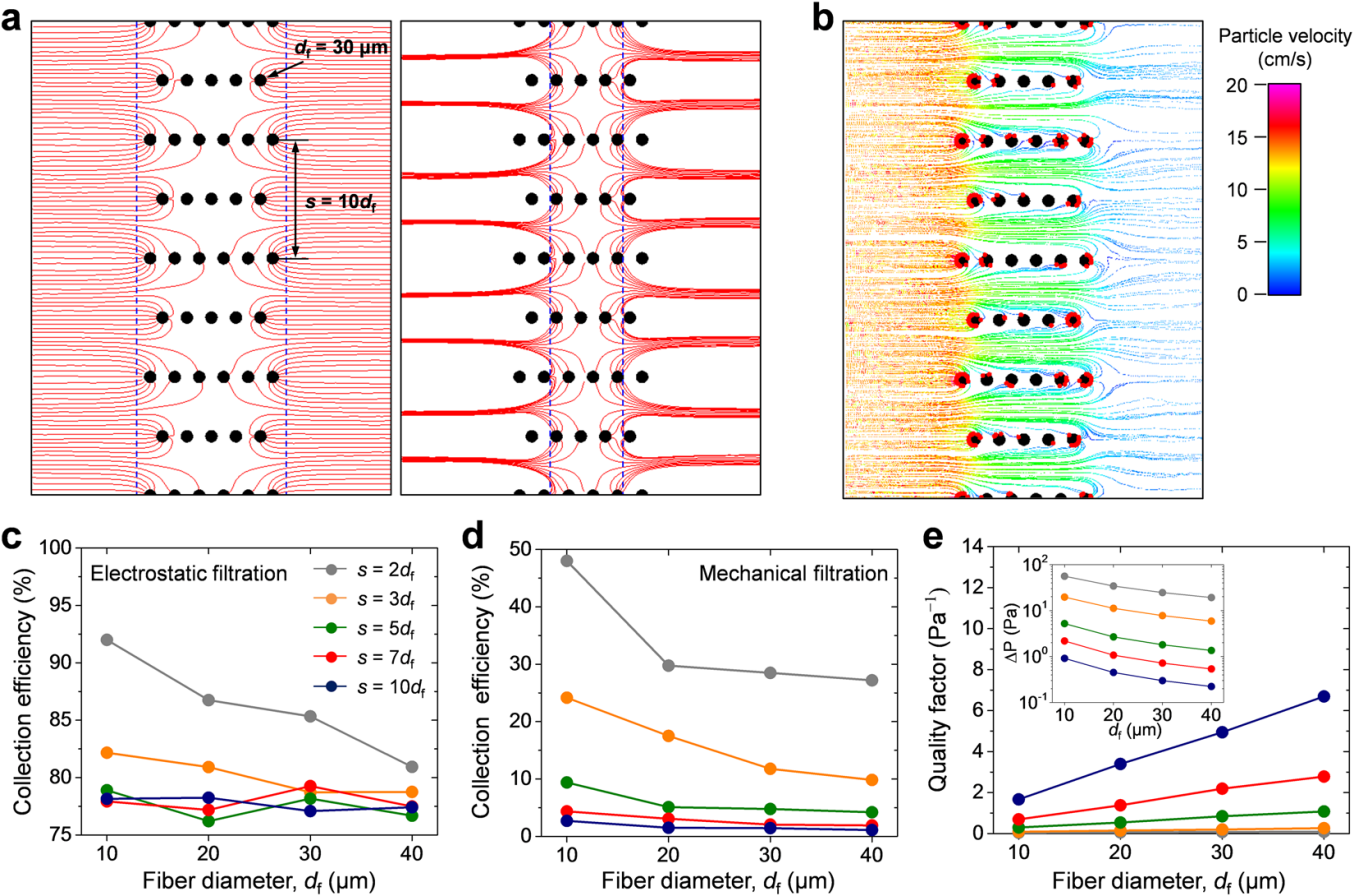
(**a**) Electric field lines produced ahead of the outermost fibers (left) and inner fibers (right). The staggered fibers have values of *d*_f_ = 30 μm and *s* = 10*d*_f_. (**b**) Particle trajectories for charged particles (*d*_p_ = 0.3 μm) when the electrostatic effect was considered. (**c**‒**e**) Numerical calculations of collection efficiency, pressure drop, and quality factor for various fiber structures: (**c**) electrostatic collection efficiency, (**d**) mechanical collection efficiency, and (**e**) quality factor. The inset shows the calculation results of the pressure drop.

Figure 6a presents the electric field lines obtained at positions equi-spaced on the dashed lines for the case of *s* = 10*d*_f_. Some of the electric field lines ahead of the outermost fibers propagated to a region deep inside the fiber layers (the image on the left in Fig. 6a). Specifically, the electric fields developed throughout the inside of the fiber layers (the image on right in Fig. 6a), unlike the case when *s* = 4*d*_f_ (Fig. 5c). Though the results are not presented here, the charged particles were mainly deposited onto the first and second fiber layers when the value of *s* was smaller than 5*d*_f_, which is very similar to the results in Fig. 5f. However, more particles were electrostatically accumulated even on the inside fibers with a larger *s* value, as shown in Fig. 6b. These results highlight the possibility of a transition of surface filtration to depth filtration as the fiber packing density decreases.

Figure 6c shows the calculated results of the electrostatic collection efficiency for various combinations of *d*_f_ and *s*. The single-fiber efficiency of the Coulomb force under the influence of a uniformly charged fiber is expressed as follows^34^:

3 $${\eta }_{C}=\pi (\frac{Qq}{3{\pi }^{2}{\varepsilon }_{0}\mu {d}_{p}{d}_{f}u}).$$

Here, *Q* is the charge per unit length of a fiber, *q* is the particle charge, and *ε*_0_ is the permittivity of free space. *μ* and *u* are the viscosity and velocity of air, respectively. According to elementary electrostatic theory, the electric field at the fiber surface with a cylindrical shape has a magnitude of *E* = *Q*/(π*ε*_0_*d*_f_). Then, the *η*_C_ in equation (3) can be rewritten as the ratio of the Coulomb force (*Eq*) and Stokes drag force (3π*μd*_p_*u*). When *s* ≤ 3*d*_f_, the electrostatic collection efficiency increased with decreasing fiber size, given that the electric field strength near the fiber surface is proportional to *d*_f_^−1^. However, the fiber size did not show a meaningful correlation with the electrostatic collection efficiency when *s* ≥ 5*d*_f_. Because effective electrostatic filtration would occur in the depth direction for the case of *s* ≥ 5*d*_f_, the efficiency improvement may be cancelled out by the decrease in thickness (*t*_f_) with fiber size (*t*_f_ $\propto$ *d*_f_ for our calculations). In contrast, the mechanical collection efficiency was improved as the fiber size or fiber packing density decreased, as shown in Fig. 6d. Furthermore, when *s* ≥ 5*d*_f_, the fiber packing density did not present a significant correlation with the electrostatic collection efficiency at a certain fiber size; a higher porosity level reduces the probability that particles will be subjected to sufficient electrostatic forces to be attracted toward the fibers. Meanwhile, the Coulombic single-fiber efficiency in equation (3) could be augmented by the reduced drag force, because the increase in porosity leads to a decrease in the gas velocity inside the filter. Beyond a certain degree of interfiber spacing, the electrostatic collection performance remains similar, regardless of the fiber diameter or the filter porosity. Hence, considering the calculated pressure drop, the quality factor improved as the fiber size and porosity increased (Fig. 6e), which suggests that the use of loosely packed filter media composed of large-sized fibers would be favorable for application in an ALCF filter.

## Discussion

In summary, we introduced an ALCF filter with a low pressure drop that utilizes electrostatic precipitation technology. Because the thin metallic Al layers formed on every fiber are electrostatically charged, the collection efficiency is less affected by the electrical properties of the ALCF filter. We found that most particles are captured by the surface filtration process based on Coulombic interactions, because the electric fields are strong only near the outer filter surfaces, unlike the inner area, which is packed with equipotential fibers. The structural properties of the ALCF filters, such as their thickness, porosity, and fiber diameter, had negligible influences on the electrostatic collection efficiency. Thus, considering their pressure drops, a thinner and more porous filter is better in terms of the quality factor. From simulations of particle motions for various fiber geometries, we suggested that surface filtration can change to depth filtration depending on the fiber packing density. For the cases of *s* ≥ 5*d*_f_, the filter porosity and fiber size exhibited only slight effects on the collection efficiency; otherwise the efficiency was affected to some degree by the structural properties. However, additional systematic experiments with a wide range of filters are required to more clearly confirm the effects of fiber structure on electrostatic filtration performance outcomes. We expect that our results will be helpful to those conducting future studies toward the development of electrostatic air filters and energy-efficient air cleaning systems.

## Methods

### Preparation of the Al Precursor Ink

The Al precursor ink, AlH_3_{O(C_4_H_9_)_2_}, was synthesized by an ethereal reaction of aluminum chloride (AlCl_3_) with lithium aluminum hydride (LiAlH_4_) in dibutyl ether (O(C_4_H_9_)_2_). All of the chemicals were obtained from Sigma-Aldrich and were used as received without further purification. AlCl_3_ (15 mM) and LiAlH_4_ (53 mM) were blended with 50 mL of dibutyl ether. The mixture was heated to ~70 °C under mild agitation for ~1 h. After the complete reaction, any LiCl byproduct and unreacted precursors were filtered and carefully discarded. The overall process was conducted in a glove box under a moisture-free argon environment.

### Fabrication of the ALCF Filter

PET nonwoven filters (Daekyung Nonwovens Co., Ltd.) were washed successively with deionized water, ethanol, and isopropyl alcohol for 10 min each in an ultrasonic bath. Then, the cleaned PET filters were dried overnight at 85 °C in an electric oven to eliminate moisture adsorbed onto the fiber surfaces before the SD process. The dried filters were immersed into a catalytic solution of Ti(O-*i*-Pr)_4_ for 30 min under mild shaking, followed by additional drying overnight at room temperature. After drying, the catalytically treated filters were dip-coated in the Al precursor ink for a certain time and fully dried at room temperature. All the coating procedures were carried out in a dry glove box filled with argon gas.

### Filtration test

KCl particles with diameters of 30–410 nm were generated by nebulizing a KCl solution (1.0 g/L in deionized water) with a one-jet collision nebulizer (Mesa Labs Inc.) at an airflow rate of 2.0 L/min. The nebulized aerosol was passed through a diffusion dryer to eliminate moisture and was diluted with clean dry air (volume ratio of 1:6). Before being introduced to the filtration device, the particles were electrically neutralized using a soft X-ray source (Aerosol Neutralizer 4530, HCT). The particle size profiles were obtained using a scanning mobility particle size (SMPS) system consisting of a differential mobility analyzer (DMA 3081, TSI) and a condensation particle counter (CPC 3771, TSI). The filtration velocity was fixed at 5 cm/s in all the experiments. The collection efficiency measurements for each condition were repeated more than three times.

### Characterization

The surface morphology and microstructure of the ALCF filters were investigated using field-emission scanning electron microscopy (MIRA3 LM, TESCAN). The energy-dispersive spectroscopy mapping results were obtained using a scanning electron microscope (Magellan 400, FEI). The sheet resistance of the filters was measured using a four-probe van der Pauw method (FPP-HS8, DASOLENG). The pressure differences across the filter were measured by a digital pressure gauge (FCO332, Furness Controls) with a detection range of 100 Pa.

### Numerical simulation

The flow field, electric field, and particle trajectory were simulated using a commercial computational fluid dynamics solver, CFD-ACE+, developed by the ESI group. The laminar flow field was obtained from the continuity and Navier Stokes equations under the assumption of an incompressible and steady-state fluid flow:

4 $$\nabla \cdot {\bf{u}}=0,$$

5 $$\rho {\bf{u}}\cdot \nabla {\bf{u}}=-\,\nabla p+\mu {\nabla }^{2}{\bf{u}},$$

where **u** and *ρ* are the velocity vector and density of air, respectively. The electric field around the fibers was calculated from the Laplace equation:

6 $${\nabla }^{2}V=0,$$

7 $${\bf{E}}=-\,\nabla V,$$

where *V* is the electric potential and **E** is the electric field vector. The particle trajectory was obtained by solving the equation of particle motion as follows^31,35^:

8 $${m}_{p}\frac{d{{\bf{u}}}_{p}}{dt}=\frac{3\pi \mu {d}_{p}}{{C}_{c}}({\bf{u}}-{{\bf{u}}}_{p})+ne{\bf{E}}+{m}_{p}\sqrt{\frac{216}{\pi }\frac{\mu kT}{{C}_{c}{\rho }^{2}{d}_{p}^{5}{\rm{\Delta }}t}}{\bf{G}}.$$

9 $${C}_{c}=1+\frac{\lambda }{{d}_{p}}[2.34+1.05\,\exp \,(-0.39{\textstyle \tfrac{{d}_{p}}{\lambda }})].$$

Here, **u**_p_ and *m*_p_ are the velocity vector and mass of the particle, respectively, and the particle shape was assumed to be spherical. The terms on the right-hand side of equation (5) denote the Stokes drag force, Coulomb force, and Brownian force acting on a particle, respectively. **G** is a Gaussian random number bounded by −1 and +1. *T* and *k* are the absolute temperature and Boltzmann constant, respectively. *C*_c_ is the Cunningham slip correction factor and *λ* is the mean free path of air.

The value of *n* was estimated based on the combined charging rate model proposed by Lawless^33^, which considers both diffusion charging and field charging simultaneously:

10 $$\frac{dv}{d\tau }=\frac{{k}_{p}w}{4}{(1-\frac{v}{{k}_{p}w})}^{2}+f(w)\,{\rm{for}}\,-\,{k}_{{\rm{p}}}w\le v\le {k}_{{\rm{p}}}w,$$

11 $$\frac{dv}{d\tau }=f(w)\frac{v-{k}_{p}w}{\exp (v-{k}_{p}w)-1}\,{\rm{for}}\,v > {k}_{{\rm{p}}}w,$$

12 $$\frac{dv}{d\tau }=-\,v+f(w)\frac{-\,v-{k}_{p}w}{\exp (\,-\,v-{k}_{p}w)-1}\,{\rm{for}}\,v < -\,{k}_{{\rm{p}}}w.$$

The dimensionless particle charge (*v*), electric field (*w*), and time (*τ*) are given by *ν* = *ne*^2^/(8π*εd*_p_*kT*), *w* = *d*_p_*E*/(2*kT*/*e*), and *τ* = (*eN*_i_*Z*_i_*t*)/*ε*, respectively, where *ε* is the permittivity of air. The fractional area of the particle obtaining the full diffusion current is expressed as *f*(*w*) = (*w* + 0.475)^−0.575^ for *w* ≥ 0.525 and *f*(*w*) = 1 otherwise. *k*_p_ is related to the particle permittivity *ε*_p_ by the following equation: *k*_p_ = 1 + 2(*ε*_p_ − 1)/(*ε*_p_ + 2). All the parameter values used in the numerical calculation are listed in Table S1. The boundary conditions for the flow field and electrostatic electric field are explained in Supplementary Information (Fig. S6).

### Data availability statement

The data that support the findings of this study are available from the corresponding author upon a reasonable request.

## Electronic supplementary material

Supplementary Information
