# Supplementary material for: Al-Coated Conductive Fiber Filters for High-Efficiency Electrostatic Filtration: Effects of Electrical and Fiber Structural Properties
Source: Sci Rep. 2018 Apr 10;8:5747. doi: 10.1038/s41598-018-23960-9 (PMC5893619; doi:10.1038/s41598-018-23960-9)
Supplement: Supplementary file 1 — Supplementary Information [file 41598_2018_23960_MOESM1_ESM.pdf]

Supplementary Information

Al-Coated Conductive Fiber Filters for High-Efficiency Electrostatic Filtration: Effects of Electrical and Fiber Structural Properties

Dong Yun Choi[1,†], *Eun Jeong An*[1,2,†], *Soo-Ho Jung*[1], *Dong Keun Song*[3], *Yong Suk Oh*[4], *Hyung Woo Lee*[2,*], *and Hye Moon Lee*[1,5,*]

[1]Powder & Ceramics Division, Korea Institute of Materials and Science, Changwondaero 797, Seongsan-gu, Changwon 51508, Korea.

[2]Department of Nano Fusion Technology, Pusan National University, 2 Busandaehak-ro 63beon-gil, Geumjeong-gu, Busan 46241, Korea.

[3]Environment and Energy Systems Research Division, Korea Institute of Machinery and Materials, Gajeongbuk-ro 156, Yuseong-gu, Daejeon 34103, Korea.

[4]Department of Mechanical Engineering, Korea Advanced Institute of Science and Technology, 291 Daehak-ro, Yuseong-gu, Daejeon 34141, Korea.

[5]Alink Co. Ltd., Chanwondaero 797, Seongsan-gu, Changwon 51508, Republic of Korea

[†]These authors contributed equally to this work.

[*]Correspondence and requests for materials should be addressed to H.M.L. (hyelee@kims.re.kr) or H.W.L. (LHW2010@pusan.ac.kr)

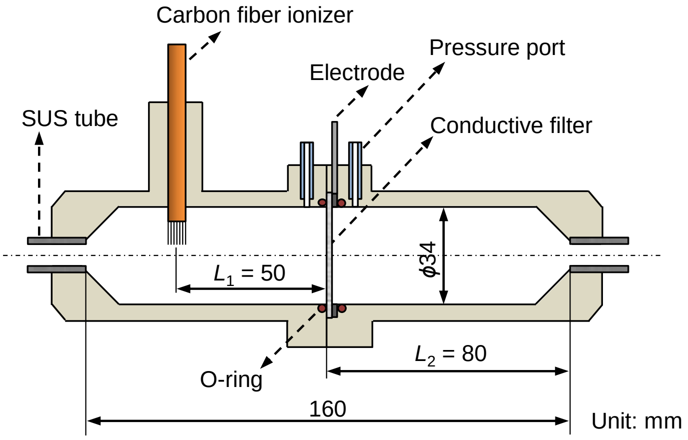

Figure S1. Structure of the electrostatic filtration device consisting of a carbon fiber ionizer and the single ALCF filter. All dimensions are in mm.

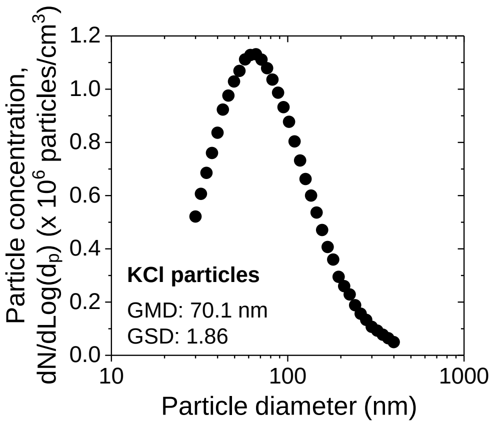

Figure S2. Size distribution of the test KCl nanoparticles generated from a nebulizer.

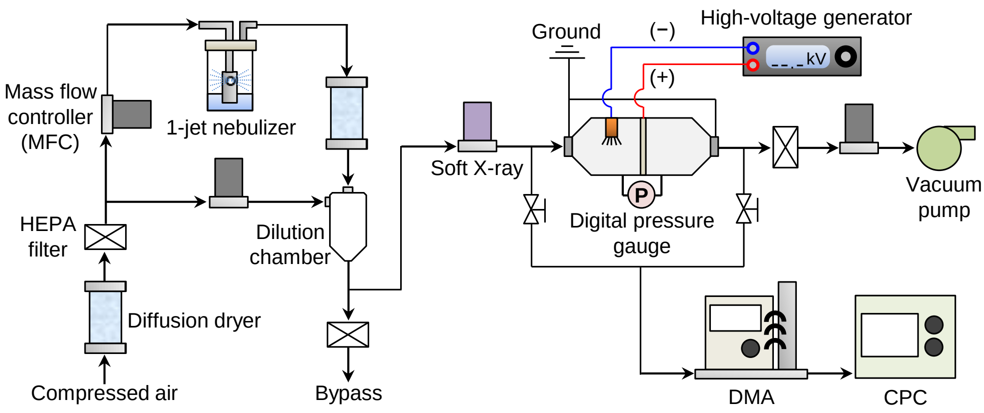

Figure S3. Schematic diagram of the experimental setup used for the measurement of the particle collection efficiency.

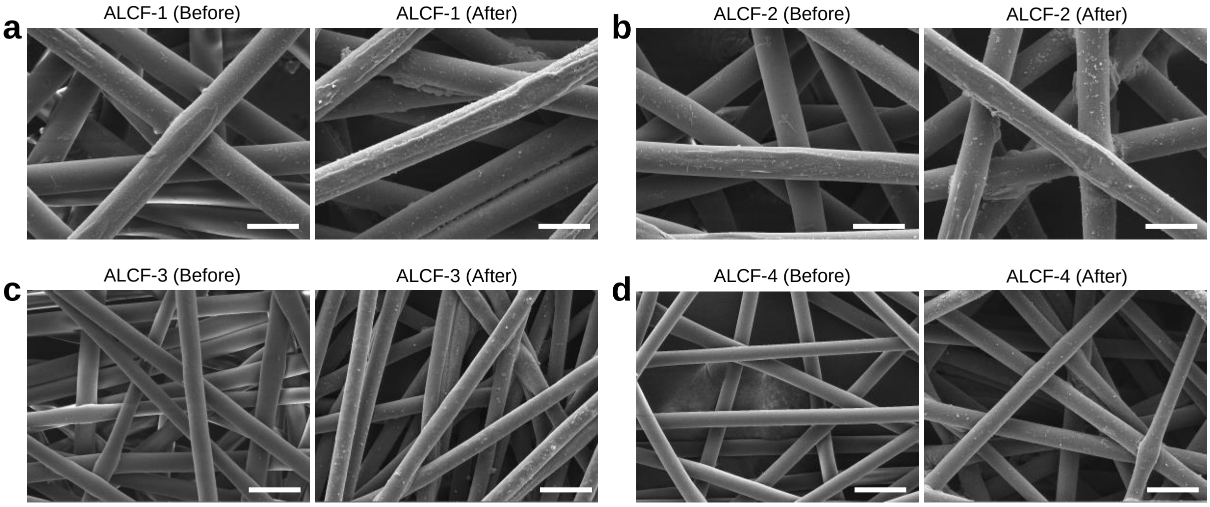

Figure S4. (a) SEM images showing the fiber microstructures of filters with different fiber structures before and after the SD process. The scale bars are 50 μm.

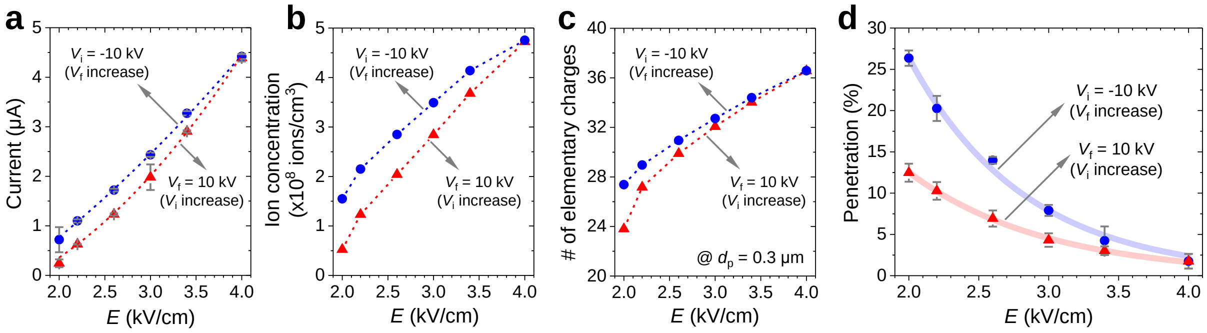

Figure S5. (a) Current curves as a function of the electric field strength (E) between the ionizer and the ALCF filter. V_f was increased from 0 to 10 kV at a constant V_i of −10 kV (blue circles), and V_i was changed from 0 to −10 kV at a constant V_f of 10 kV (red triangles). (b) Plot of the calculated ion concentrations at each condition of E. (c) Plot of the number of elementary charges acquired by a particle 0.3 μm in size according to the E strength. (d) Particle penetration characteristics depending on the conditions of V_i and V_f at identical E strengths.

The ion generation by the carbon fiber ionizer depends on the given electric potential and the surrounding electric field and consequently influences the charging rate of the particles and the filtration performance of the ALCF filter. We examined the particle charging and particle penetration characteristics depending on the magnitudes of the ionizer voltage (V_i) and the

filter voltage (V_f). Figure S5a shows the current (I) curves as a function of the electric field strength (E) between the ionizer and the filter. The generated ions were collected on an ALCF filter and their current was measured. When V_i was set to −10 kV (blue circles), the current was higher compared to the condition of the constant V_f of 10 kV (red triangles) at each value of E. This result confirms that N_i was affected by the electric potential of the ionizer as well as the electric field developed around the ionizer. N_i was calculated from the measured I using the equation of $N_i = I/(AZ_iEe)$, where A is the filter area, Z_i is the ion mobility, and e is the elementary charge[1]. The calculated values of N_i are plotted in Fig. S5b as a function of E. N_i became saturated gradually as E increased, but the increase of I became higher because the ions were transferred at a velocity of Z_iE. Fig. S5c presents the values of n for a 0.3 μm particle calculated by the combined charging rate model[2]. The increases in the value of n slowed down as E was increased, and the value of n when V_i = −10 kV was higher than when V_f = 10 kV. However, the differences between the values of N_i at a given E did not make a great gap in the results of n.

The particle penetration results for each case of V_i = −10kV and V_f = 10 kV are given in Fig. S5d, and they exhibited an exponential decrease with an increase in E. Interestingly, the particle penetration for V_f = 10 kV was superior to that for V_i = −10 kV despite the smaller value of n. The lower penetration rate appears to be due to the backward deposition of the outgoing particles onto the back side of the ALCF filter by the higher E formed downstream of the filter.

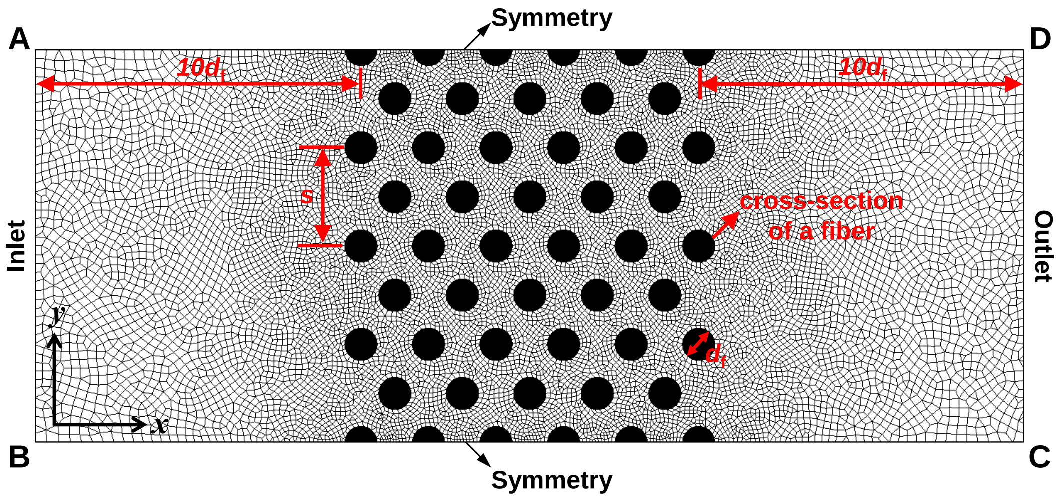

Figure S6. A 2D calculation domain used for the simulations.

The flow field, electric field, and particle trajectories were numerically calculated from a commercial computational fluid dynamics solver, CFD-ACE+, developed by ESI group. A 2D model with quadrilateral meshes was built to describe the particle motions around an array of staggered fibers. Figure S6 shows the model geometry corresponding to the conditions of a fiber diameter (d_f) of 30 μm and interfiber spacing (s) of 3d_f. The continuity equation and Navier-Strokes equations are solved with the assumptions of the steady-state incompressible flow. The boundary conditions for the flow field are given as:

$$u_x = u_0 \ \& \ u_y = 0 \qquad \text{at the inlet (line AB) ,} \tag{S1}$$

$$p = p_{atm} \qquad \text{at the outlet (line CD),} \tag{S2}$$

$$u_y = 0 \ \& \ \frac{\partial u_x}{\partial y} = 0 \qquad \text{along the line BC and the line AD,} \tag{S3}$$

$$u_x = u_y = 0 \qquad \text{at the fiber surface (non-slip condition),} \tag{S4}$$

where, u_x and u_y are the gas velocities in the x direction and y direction, respectively. p is pressure variable, and p_{atm} is atmospheric pressure. For all calculations, we assumed that the face velocity in front of the filter is constant with a value of u_0 = 5 cm/s.

The electric field in the computational domain was solved by using the Laplace equation. Constant electric potentials were given at the inlet, outlet, and all fiber surfaces. And, other boundary conditions were set to symmetry. The value of the electric potential at the inlet was approximated by the linearity assumption of the electric potential difference between the ionizer and the ALCF filter. Similarly, a linear potential difference between the ALCF filter and the grounded SUS tube was assumed in order to set the value of the electric potential at the outlet.

$$V = V_f - 10 d_f \frac{V_f - V_i}{L_1} \quad \text{at the inlet (line AB) ,} \tag{S5}$$

$$V = V_f - 10 d_f \frac{V_f}{L_2} \quad \text{at the outlet (line CD),} \tag{S6}$$

$$V = V_f \quad \text{at the fiber surface,} \tag{S7}$$

$$\frac{\partial V}{\partial y} = 0 \quad \text{along the line BC and the line AD.} \tag{S8}$$

Here, the V_i is the applied voltage to the carbon fiber ionizer and the V_f is the applied voltage to the ALCF filter. As shown in Figure S1, L_1 is the distance between the ionizer and the ALCF filter, and L_2 is the distance between the ALCF filter and the grounded SUS tube. For all calculations, V_f and V_i were fixed to 10 kV and −10 kV, respectively.

Table S1. Parameter values used in the numerical calculations.

Parameter	Value	Parameter	Value
Particle density (KCl), ρ_p	1.98 g/cm^3	Ion concentration, N_i	4.8×10^{14} ions/m^3
Particle relative permittivity, ε_p	4.81	Negative ion mobility, Z_i	1.6×10^{-4} m^2/Vs
Particle diameter, d_p	0.3 μm	Elementary charge, e	1.6×10^{-19} C
Viscosity of air, μ	1.81×10^{-5} Pa s	Permittivity of air, ε	8.85×10^{-12} C^2/Nm^2
Mean free path of air, λ	0.066 μm	Boltzmann constant, k	1.3086×10^{-23} J/K

References

1. Lin, G.-Y., Chen, T.-M. & Tsai, C.-J. A Modified Deutsch-Anderson Equation for Predicting the Nanoparticle Collection Efficiency of Electrostatic Precipitators. *Aerosol Air Qual. Res.* **12**, 697-706 (2012).
2. Lawless, P. A. Particle charging bounds, symmetry relations, and an analytic charging rate model for the continuum regime. *J. Aerosol Sci.* **27**, 191-215 (1996).
